# Hereditary transthyretin amyloidosis: a myriad of factors that influence phenotypic variability

**DOI:** 10.1007/s00415-024-12509-8

**Published:** 2024-06-22

**Authors:** Estefânia Carvalho, Andreia Dias, Teresa Coelho, Alda Sousa, Miguel Alves-Ferreira, Mariana Santos, Carolina Lemos

**Affiliations:** 1 Instituto de Investigação e Inovação Em Saúde (i3S), University of Porto, Porto, Portugal; 2 Instituto de Ciências Biomédicas Abel Salazar (ICBAS), University of Porto, Porto, Portugal; 3 Unidade Corino de Andrade (UCA), Centro Hospitalar Universitário de Santo António (CHUdSA), Porto, Portugal; 4 Center for Preditive and Preventive Genetics (CGPP), Institute for Molecular and Cell Biology (IBMC), Instituto de Investigação e Inovação Em Saúde (i3S), University of Porto, Porto, Portugal; 5 Institute for Molecular and Cell Biology (IBMC), Instituto de Investigação e Inovação Em Saúde (i3S), University of Porto, Porto, Portugal

**Keywords:** Amyloidosis, Transthyretin, Phenotypic variability, Diagnosis

## Abstract

Hereditary transthyretin-related amyloidosis (ATTRv amyloidosis) is a rare and progressively debilitating disease characterized by the deposition of transthyretin (TTR) amyloid fibrils in various organs and tissues, most commonly in the heart and peripheral nerves. This pathological deposition can lead to significant organ dysfunction and, ultimately, organ failure. ATTRv amyloidosis exhibits a broad range of clinical presentations, from purely neurological symptoms to purely cardiac manifestations, as well as mixed phenotypes which result from both neurological and cardiac implications. This wide phenotypical spectrum realistically challenges disease diagnosis and prognosis, especially in individuals without or with an unknown family history. Multiple factors are thought to contribute to this variability, including genetic, epigenetic, and even environmental influences. Understanding these factors is crucial, as they can significantly affect disease expression and progression. This review aims to summarize each of these contributing factors, to help elucidate the current knowledge on the phenotypical variability of ATTRv amyloidosis.

## Introduction

First described in Portugal, in 1952 [1], hereditary transthyretin-related amyloidosis (ATTRv amyloidosis—formerly “Familial Amyloid Polyneuropathy”) is a fatal, progressive, autosomal-dominant disorder caused by variants in the transthyretin (*TTR*) gene [2].

*TTR*, located on chromosome 18, encodes transthyretin (TTR—formerly “prealbumin”), a homotetrameric protein [3] produced predominantly in the liver, though small amounts are synthesized in the choroid plexus, retinal epithelium, and pancreatic islets [4–6]. Its primary functions include the transport of thyroxine and the retinol-binding protein bound to retinol, although other potential roles have been discovered [7].

**Fig. 1:**
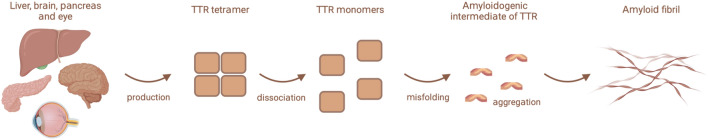
Schematic representation of TTR amyloidogenesis. Image constructed with BioRender and adapted from [10]

It is widely accepted that protein destabilization is a crucial factor behind amyloid fibril formation [8]. In such case, variants in the *TTR* gene lead to protein destabilization and dissociation of its tetramer structure into dimers and subsequently into monomers. These monomers misfold and form the amyloidogenic intermediate of TTR that then aggregate into amyloid fibrils which deposit in several tissues and organs, mainly in nerves, heart, kidneys, and eyes (Fig. 1). The deposits in these organs and tissues lead to organ dysfunction and possible organ failure giving rise to a vast array of symptoms [8, 9].

However, it is important to mention that TTR proteolysis also has been extensively studied as a mechanism that can drive amyloid formation [11]. This is due, in large part, to the presence of C-terminal TTR fragments in ex vivo TTR amyloid deposits regardless of the TTR variant [12].

Importantly, wild-type TTR is also responsible for a relatively common, aging-related, nonhereditary type of amyloidosis termed senile systemic amyloidosis or wild-type TTR amyloidosis (ATTRwt amyloidosis), where the heart is the main affected organ [13, 14].

Nonetheless, regarding ATTRv amyloidosis, one of its most striking aspects is the wide range of clinical presentations that can occur. There are more than 140 *TTR* variants (most of which are pathogenic), and each variant can cause a different pattern of symptoms, disease severity, and age of onset [15]. The *TTR* Val30Met variant is one of the most prevalent variants worldwide (possibly only trumped by the Val122Ile variant and its occurrence is in 3–4% of African Americans [16]) and is responsible for the high prevalence of ATTRv amyloidosis in endemic regions such as Portugal, Sweden, and Japan [17–19].

The most common symptom of ATTRv amyloidosis, its “hallmark”, is a progressive peripheral sensory–motor neuropathy, which typically begins in the feet and hands and can cause tingling, numbness, pain, and weakness. Other common manifestations include autonomic dysfunction, cardiomyopathy, nephropathy, and gastrointestinal and ophthalmological abnormalities.

Autonomic symptoms are common and can involve various systems, including the cardiovascular, gastrointestinal, and genitourinary systems and symptoms include orthostatic hypotension, erectile dysfunction, sweating abnormalities, and recurrent urinary tract infections. Cardiovascular involvement can lead to cardiomyopathy, which can cause arrhythmias, atrial fibrillation, and heart failure. Gastrointestinal dysfunction can lead to symptoms such as nausea, vomiting, diarrhea, constipation, and early satiety. These symptoms can be particularly debilitating and can contribute to malnutrition and weight loss. Renal involvement, although less common than neurological or cardiac symptoms, may also occur, causing proteinuria and kidney failure. In addition, patients with ATTRv amyloidosis may also develop ocular symptoms such as dry eyes, abnormal conjunctival vessels, papillary abnormalities, and glaucoma [20–22].

Given this immense phenotypical variability, patients may present symptoms that often overlap with other conditions, being frequently misdiagnosed with diseases such as idiopathic axonal polyneuropathy, chronic inflammatory demyelinating polyneuropathy (CIDP), or heart failure and might not be correctly diagnosed until later in the disease course [20]. This variability makes ATTRv amyloidosis difficult to diagnose, especially in older patients or those without a known family history [23, 24].

Understanding the biological reason behind symptom variability in ATTRv amyloidosis is extremely important since it can provide insights into the disease mechanisms and reveal potential targets for treatment.

**Fig. 2:**
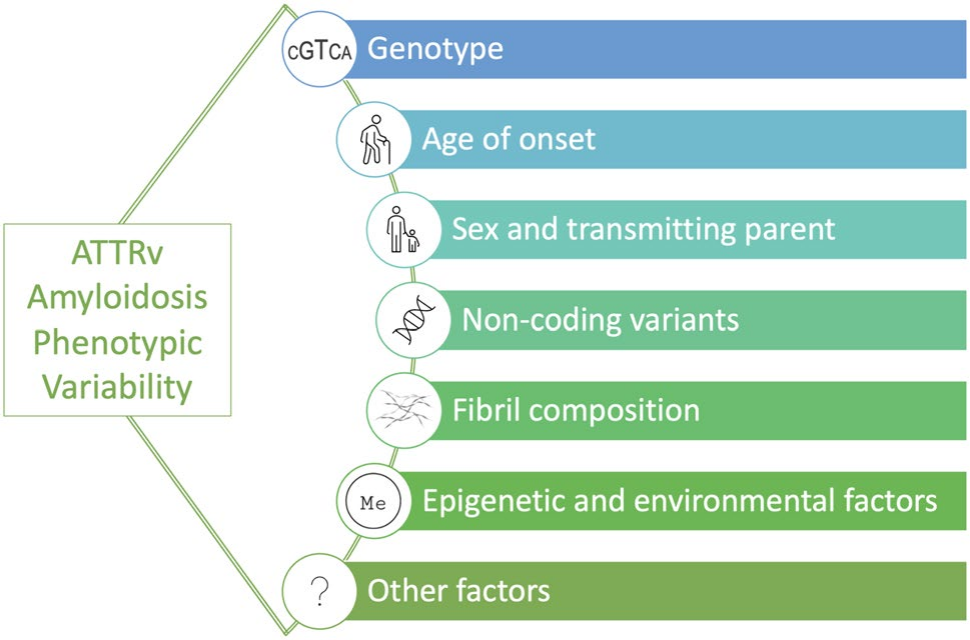
Factors that potentially influence ATTRv amyloidosis phenotypic variability

In this review, we discuss the current knowledge on factors that might influence disease outcome and help explain phenotype variability (Fig. 2). A summarized overview of this review is presented in Table 1.

**Table 1:** Overview of factors discussed in this review that influence ATTRv amyloidosis phenotype

Factor	Description	Impact on phenotype
Genotype	Over 140 *TTR* variants, most are pathogenic; specific variants are linked to distinct phenotypes (neuropathic, cardiac, mixed)	Determines clinical presentation, disease severity, and affected organs
Age of onset	Varies widely: early onset (< 50 years) and late onset (> 50 years) Independent clusters show significant divergence	Influences disease course and severity. Early onset is typically associated with neuropathy and late onset with a cardiac phenotype
Sex and transmitting parent	Males tend to have earlier onset and more severe cardiomyopathy Maternal inheritance shows larger anticipation	Sex differentially impacts prevalence and severity; maternal transmission linked to earlier onset
Non-coding variants	Variants in regulatory regions affect gene expression	Potentially influence severity, symptoms, and age of onset
Fibril composition	Type A fibrils (C-terminal fragments and full-length TTR) vs. Type B fibrils (intact TTR)	Type A associated with later onset and cardiac involvement; Type B with early onset and little to no cardiac issues
Epigenetic factors	DNA methylation; histone modification; miRNAs	Influence gene expression and disease variability
Environmental factors	Factors such as organic solvent exposure, anesthesia history, gut microbiota	Possible contribution to amyloidogenesis and disease variability
Other factors	Monoallelic expression; somatic mosaicism	May explain variability in onset and severity; impacts diagnostic accuracy

## Genotype

As stated above, *TTR* has over 140 variants described and most of them are pathogenic. Although the same variant can be associated with an array of symptoms, some are more associated with a specific phenotype than others. This is known as the genotype–phenotype correlation. Generally, clinical presentation can be predominantly neuropathic, predominantly cardiac, or mixed (exhibiting both polyneuropathy and cardiomyopathy) [25]. The most common *TTR* variants and their predominant phenotype are presented in Table 2.

**Table 2:** Most common TTR variants and their main geographic focus and predominant phenotype

Variant	Main geographic focus	Predominant phenotype	References
Val30Met	Portugal, Brazil, Spain (regions of mostly early-onset cases)	Neuropathic (with more autonomic dysfunction)	[26–28]
	Japan, Sweden, USA, Italy (regions of mostly late-onset cases)	Neuropathic (more severe and with cardiac involvement)	
Val122Ile	West Africa, USA, Caribbean, UK	Cardiac	[29–31]
Thr60Ala	UK, Ireland	Cardiac	[32, 33]
Glu89Gln	Italy, Bulgaria	Mixed	[34, 35]
Ser50Arg	Mexico	Neuropathic	[36, 37]
Phe64Leu	Italy	Mixed	[38, 39]
Ile68Leu	Italy	Cardiac	[40, 41]
Leu111Met	Denmark	Cardiac	[42, 43]
Ser77Tyr	Israel, France	Mixed	[44–46]

Despite the high prevalence of TTR V30M, data indicates that patients’ survival rate is quite high, especially when compared to other variants, such as Thr60Ala, Val122Ile, or even wild-type TTR (wtTTR), which are generally associated with poorer prognoses. Intriguingly, these variants are typically linked to a more cardiac-type disease [47].

Pathogenic *TTR* variants are generally amyloidogenic, though most of the variants reported were identified in a small group of patients and their pathogenicity is not clear. However, some variants have been reported to have higher or lower amyloidogenicity. For instance, although rare, TTR variant Leu55Pro is notably aggressive, exhibiting a high propensity for protein aggregation. It is associated with rapid disease progression of mixed symptoms and early onset, with some individuals experiencing initial symptoms during childhood [48–51].

In contrast, *TTR* variant Glu61Lys, a rare variant so far only identified in late-onset patients, has low amyloidogenicity similar to that of wild-type *TTR* [52]. Interestingly, in patients carrying this variant, sural nerve biopsy showed no amyloid deposits, although significant loss of myelinated fibers was detected. Deposits were, however, present in muscle, salivary gland, and heart biopsies [52–54].

Additionally, some non-pathogenic variants (Thr119Met and Arg104His) can have a protective effect in compound heterozygotes carrying the Val30Met variant. The simultaneous occurrence of these protective variants and Val30Met results in the absence of symptoms or a delayed form of the disease [55, 56]. It is thought that these variants increase TTR tetramer stability and thus increase its resistance to dissociation [57, 58].

The full list of all known *TTR* variants is presented in the “Variants in Hereditary Amyloidosis” database (http://amyloidosisvariants.com).

The specific pathogenic *TTR* variant is a significant factor in the clinical variability of ATTRv amyloidosis. Each variant has a unique impact on clinical presentation and disease course. However, the amino acid substitutions alone do not completely explain the variability in disease penetrance, pathology, and clinical course. This is evident especially in cases of monozygotic twins that share the same variant, but have very different clinical presentations [59–62].

The genotype of ATTRv amyloidosis significantly influences its clinical presentation, with over 140 known *TTR* variants showing diverse pathogenic effects. Certain variants are strongly associated with specific phenotypes—neuropathic, cardiac, or mixed. Additionally, some non-pathogenic variants can mitigate the severity of symptoms in individuals with the Val30Met variant. Despite the clear genotype–phenotype correlations, variability in disease expression remains, highlighting the complexity of ATTRv amyloidosis and the need for further research to fully understand these genotype influences.

## Age of onset

In ATTRv amyloidosis, age of onset, the age at which a person first experiences symptoms, varies greatly and can range from early adulthood to old age [63]. Carriers of *TTR* variants can even remain asymptomatic their whole life [64]. Age of onset is generally divided into two groups, early and late onset. Early-onset individuals exhibit their first symptoms before the age of 50, whereas late-onset patients only experience their first symptoms after that age [65, 66]

Regarding the *TTR* Val30Met variant, despite having the same genotype, the independent clusters of endemic regions have substantial divergence in the age of onset. In Portugal, the average age of onset is 33.5 years and 87% of patients show symptoms before the age of 40 [67]. In Japan, three endemic foci have been reported, namely, in the Nagano, Kumamoto, and Ishikawa prefectures and the mean ages of onset are 33.1, 35.6, and 62.9 years of age, respectively [18, 66, 68]. In non-endemic areas in Japan, the age of onset is typically higher [69]. The Swedish endemic foci, which are around the towns of Skellefteå and Piteå, are regarded as being of late onset, as the mean age of symptom presentation is 54.4 and 58.8 years of age, respectively [70].

Origin from endemic areas, as opposed to non-endemic regions, is generally associated with higher penetrance and lower age of onset, with the exception of Swedish endemic regions that present low penetrance and late onset [71, 72]. Noteworthy, non-Val30Met variants are generally regarded as having a later onset [73].

Still concerning the Val30Met variant, it is also noteworthy to mention the phenomenon of anticipation. Anticipation is regarded as the decrease of age of onset within each generation [74]. Parents affected with ATTR Val30Met amyloidosis and a late-onset presentation frequently have early-onset children; however, the opposite has yet to be described. Anticipation is currently considered a true biological phenomenon in ATTR Val30Met amyloidosis and has been reported in several clusters [74–78].

As stated in Table 1, individuals with the Val30Met variant and early onset typically have a less severe disease course with neuropathy and considerable autonomic dysfunction. In contrast, late-onset Val30Met patients have more severe symptoms with less autonomic dysfunction and moderate to severe cardiac involvement [27, 73].

Since the age of onset has a great variability and seems to influence the phenotype and disease course, it is hypothesized that genetic modifiers may explain the age of onset variability. Identifying modifier genes helps to better understand the underlying mechanisms involved in the pathophysiology of diseases and to find new targets for therapeutic intervention [79]. Thus, studies have been performed to access the existence of genetic modifiers of age of onset in ATTRv amyloidosis.

The Val30Met variant is probably the most studied regarding genetic modifiers given its wide range of age of onset and the different clinical presentations between early- and late-onset individuals. Several associations have made between genetic variants and the age of onset of ATTR Val30Met amyloidosis and these are summarized in Table 3.

**Table 3:** Potential genetic modifiers of age of onset in ATTR Val30Met amyloidosis described in the literature

Gene	Variant/SNP ID	Associated with	Studied population	Notes	References
*C3*	*C3F* (“fast” polymorphism)	Early onset	Swedish	–	[80]
Combined effect: *APCS* (2 variants)	rs6689429, rs2808661	Early onset	Portuguese	–	[81]
Combined effect: *APCS* (1 variant); *RBP4* (2 variants)	rs6689429; rs7091052, rs28383574	Late onset		–	
*C1QA*	rs172378	Early onset	Greek–Cypriot	Lost significance after controlling for family	[82]
*ApoE*	Genotype ε2/ε3	Early onset		–	
*C1QC*	rs9434	Late onset		–	
*APCS*	rs28383573	Late onset	Portuguese	–	[83]
*RBP4*	rs7094671	Late onset		–	
*RBP4*	rs11187545	Early onset		–	
*AR*	In the female group: rs5919392, rs2361634, rs5965433 In the male group: rs5919393, rs17217069, rs2361634	Early onset		Located in the X chromosome, so analyses were stratified according to sex	
*AR*	In the female group: rs5919393	Late onset			
*NGAL*	rs3780836	Early onset	Portuguese	–	[84]
*MEK1*	rs8039880, rs11630608, rs745796	Early onset		–	
*HSP27*	rs11769502	Early onset		–	
*YWHAZ*	rs17365305	Early onset		–	
*BGN*	In the female group: rs22694049	Late onset		Located in the X chromosome, so analyses were stratified according to sex	
*MEK1*	rs16949939	Late onset		–	
*MEK2*	rs1823059	Late onset		–	
*ATXN2*	Presence of at least 1 allele longer than 22 CAG repeats	Early onset	Portuguese	–	[85]
*C1Q*	rs665691, rs158761, rs172378, rs672693, rs9434, rs294180	Early onset	Cypriot	–	[75]
*C1QA*	rs201693493, rs149050968	Late onset	Portuguese	–	[86]
*C1QC*	rs200952686	Late onset		–	
*C1QC*	rs2935537, rs201241346	Early onset		–	

The age of onset in ATTRv amyloidosis varies widely, ranging from early adulthood to old age, and significantly impacts the disease phenotype and course. The Val30Met variant displays considerable regional differences in the age of onset, potentially influenced by genetic modifiers. Despite research advances, variability in age of onset remains to be explained. Understanding the mechanisms behind this variability will help predict patients’ age of onset, aiding in genetic counseling, and can also be important in determining prognosis and in developing new therapeutic strategies.

## Sex and transmitting parent

Sex is an important modifier of disease, as it plays a role in epidemiology, disease pathogenesis, clinical presentation, and therapy response. These differences are ultimately attributed to the genetic heterogeneity of sex chromosomes and the hormonal influences that create molecular differences between males and females [87, 88]. In ATTRv amyloidosis, sex has also been implicated.

According to the Transthyretin Amyloidosis Outcome Survey (THAOS), a worldwide registry for ATTR amyloidosis, the symptomatic carriers of the disease are predominantly male in both wild-type ATTR amyloidosis (ATTRwt amyloidosis or senile systemic amyloidosis) and ATTRv amyloidosis [73]. Regarding the Val30Met variant, it has also been reported that males tend to have earlier onsets and/or higher disease risks than females [77, 78, 89–91].

Another study from THAOS on 4050 ATTRv amyloidosis carriers (2790 symptomatic and 1260 asymptomatic) showed that a higher proportion of men had cardiac involvement and that a predominantly cardiac phenotype was more common in males than females [92]. In fact, men represented a staggering 72% of patients that had ATTRv amyloidosis with cardiomyopathy. On the other hand, women represented a higher proportion of the individuals in the asymptomatic group. Although disease prevalence in males was overall generally higher than in females, it was found to be especially high in late-onset *TTR* Val30Met and non-Val30Met cardiac variant carriers. The study also showed a significant association between males and an earlier disease onset.

Another study from THAOS analyzed the *TTR* Val122Ile carriers with cardiac amyloidosis in the USA and also found increased male prevalence (75.8%) [93]. Similarly, in the UK, a study on the Val122Ile variant also showed increased male prevalence [94]. Moreover, Batra and colleagues also studied *TTR* Val122Ile carriers with cardiac amyloidosis and determined that although cardiac function and mortality rates were similar between sexes, females were significantly older at the time of diagnosis which could indicate a slower disease progression [95].

A study by Rapezzi et al. examined carriers of different *TTR* variants and reported that men had a higher prevalence of cardiomyopathy. The authors postulated that female hormones could exert a protective effect and so, in further analysis, they found that women with higher degrees of cardiomyopathy were more likely to be menopausal, which strengthened their theory [96].

Briefly, men seem to have more cardiac involvement irrespective of *TTR* variant. It has been theorized that female hormones may have a protective effect; however, the role of male hormones should also be considered as it has been reported that they can also upregulate *TTR* [97].

Nevertheless, sex-related differences are still a rather understudied topic and reports can differ especially in those with smaller cohorts.

Besides sex of the variant carrier, the sex of the parent who transmitted the variant might also have an impact on disease presentation. This factor has been mentioned in many studies regarding the Val30Met variant.

As early as 1991, two studies of Portuguese cohorts of Val30Met carriers not only reported the phenomenon of anticipation, but also observed a larger anticipation in offspring of affected mothers [89, 98]. In either case of parent transmission, men displayed an earlier onset than women, and thus sons of affected mothers had the earliest of onsets while daughters of affected fathers had the latest onsets.

Similarly, other studies, including in Swedish and Japanese cohorts, have revealed that anticipation was especially significant when the variant was inherited maternally [76, 99] and especially in mother–son pairs [77, 78].

Noteworthy, a study on the penetrance of ATTR Val30Met amyloidosis on a Swedish cohort revealed a significant association between higher penetrance of the disease and maternal inheritance of the *TTR* variant [100]. These findings are supported in other studies [77, 101].

Potential mechanisms responsible for these phenomena include parental imprinting and mitochondrial involvement. The imprinting possibility is not one much explored, but the role of mitochondrial DNA has been examined, albeit vaguely.

To understand the higher mean age of onset of Swedish and French patients, Olsson et al. investigated the mitochondrial haplogroups in Swedish and French *TTR* Val30Met carriers and controls [102]. They found that the mitochondrial haplogroups were similar between late-onset patients and controls, an expected outcome. Notably, they found that the rare mitochondrial haplogroup K appeared recurrently in early-onset patients, indicating that this haplogroup could potentially have an impact on amyloidogenesis. A subsequent study determined that this haplogroup could explain the variability seen regarding sex of the transmitting parent [103].

Similarly, mitochondrial DNA (mtDNA) copy number was assessed in Portuguese ATTR Val30Met amyloidosis patients [104]. A significantly higher mtDNA copy number was found in the asymptomatic, early-onset and late-onset groups when compared to controls, but failed to show significant differences among each other. When parent–offspring pairs where analyzed, an important increase of mtDNA copy number was seen in early-onset children compared to their late-onset parents.

To summarize, sex can significantly influence the epidemiology, clinical presentation, and disease progression in ATTRv amyloidosis. Males tend to have an earlier onset and higher prevalence of cardiac involvement, while females are more often asymptomatic. Studies suggest that female hormones might have a protective effect against cardiomyopathy. Additionally, the sex of the parent transmitting the variant also impacts disease presentation, with maternal inheritance often associated with earlier onset and higher penetrance. Potential mechanisms for these differences include parental imprinting and mitochondrial DNA, though further research is needed to fully understand these influences.

## Non-coding variants

Non-coding variants are single-nucleotide polymorphisms (SNPs) or structural variants (insertions, deletions, copy-number variants, and repeat expansions) that occur in the non-coding regions and that have gained growing importance being now considered contributors to disease due to their role in gene regulation and expression [105].

Depending on the localization of the variant, effects can include, but are not limited to, (i) alterations in the binding of transcription factors and RNA polymerase (in regulatory regions); (ii) alterations in splicing (in splicing sites); and (iii) alterations in the binding of miRNA and RNA-binding proteins (in untranslated regions). All these changes may result in RNA instability, aberrant protein isoforms, and an altered gene regulation and expression [106]. It is thus tempting to assume that non-coding variants might be a driving force behind the clinical variability of ATTRv amyloidosis.

Several studies that report non-coding variants in ATTRv amyloidosis are described in Table 4. Although the role of non-coding variants in this disease is still undetermined, together, these studies help shed light on the importance that this field might have in the better understanding of ATTRv amyloidosis.

**Table 4:** Reported non-coding variants in ATTRv amyloidosis

Type of analysis	Study sample	Objective of the study	Main findings	Main conclusions	References
Microsatellite and single-nucleotide polymorphism (SNP) haplotyping; genomic sequencing	170 Val30Met carriers + 21 familial non-carriers + 92 controls (Swedish and Portuguese)	To characterize genetic variation of the *TTR* gene and its surroundings	Identification of 10 new polymorphisms in the *TTR* untranslated regions (8 SNP and 2 insertions/deletions): identification of 13 microsatellite and 6 SNP haplotypes in Portuguese individuals, and 6 microsatellite and 4 SNP haplotypes in Swedish individuals	A non-coding region downstream the *TTR* gene of the non-carrier chromosome could exert a modulatory effect on age of onset	[107]
In silico analysis (cluster and linkage disequilibrium, haplotype, functional impact) of a 20 kb region that included *TTR*	1000 Genomes Project Database —1092 healthy individuals from 14 populations and 4 ethnic origins	To explore variants between ethnic groups that could account for ATTRv amyloidosis variability	Detection of *TTR* Val122Ile in apparently healthy African and American individuals; genetic and haplotypical differences in non-coding regions of *TTR* between Africans and non-Africans, some of which may have functional impact on the *cis*-regulatory elements of *TTR*; some non-coding variants are located in the predicted binding sites of transcription factors involved in cardiac function	Val122Ile haplotype may carry non-coding variants involved in cardiac functions, which could explain the cardiac involvement in TTR Val122Ile amyloidosis	[108]
In silico analysis (functional impact, genomic conservation, amyloidogenic propensity, transcription factor and protein binding) of a 20 kb region that included *TTR*	1000 Genomes Project Database	To analyze in silico the *TTR* gene so as to understand the pathogenesis of ATTR amyloidosis	Identification of 63 genetic variants (59 located in non-coding regions) of the *TTR* gene that could potentially have a functional impact on the gene	Variants in cis-regulatory elements of *TTR* could potentially modulate the disease phenotype	[109]
Sequencing of a 20 kb region that included *TTR*	Phenotypical analysis: 129 symptomatic Italian patients Genetic analysis: 55 symptomatic Italian patients	To evaluate how non-coding variants impact *TTR* expression	Identification of 43 SNPs and 10 insertions/deletions; strong correlation of a Val30Met haplotype with ocular involvement (haplotype contains 2 regulatory non-coding variants); considering the non-coding variants found, 3 patient clusters were identified that had specific patterns of *TTR* expression and were associated with particular phenotypes	Non-coding variants and gene expression profiles could be contributors to disease presentation	[110]
Phenome-wide association study of *TTR* and *RBP4* (using an overall and sex-stratified approach)	361,194 individuals of European descent from the UK Biobank	To assess genotype–phenotype correlations of ATTRv and ATTRwt amyloidoses	Ascertained 382 clinically relevant phenotypes related to the pathogenesis of either amyloidosis (i.e., chronic ischemic heart disease, dysphagia, bladder disorders); Identified *TTR* non-coding variants as being associated with several relevant phenotypic traits in the overall, female-specific, and male-specific analyses	*TTR* non-coding variants could play a role in the phenotypic presentation of ATTR amyloidosis patients	[111]
SNP sequencing; haplotype analysis	Portuguese individuals: 589 ATTR Val30Met carriers + 132 familial non-carriers + 189 controls	To identify genetic modifiers within or linked to *TTR* that could be associated with age of onset of ATTR Val30Met amyloidosis	Uncovered 8 SNPs at the *TTR* locus and constructed haplotypes from them; haplotype C was associated with very early onset (≤ 30 years of age) and always inherited by the non-carrier parent; allele A of intronic SNP rs72922947 was associated with early onset	Haplotype C may exert a *trans* effect on age of onset of ATTR Val30Met amyloidosis	[112]
Sequencing of the *TTR* promoter, coding regions and intron/exon boundaries; in silico analysis of variant impact on TTR splicing, transcription factors, and miRNA binding	330 ATTR Val30Met carriers from Portugal	To identify *TTR* variants that could help explain variability in age of onset of ATTR Val30Met amyloidosis	8 rare variants on the promoter and 3 on the coding and flanking regions were significantly associated with age of onset; In coding and flanking regions, 2 variants showed association with a late symptom onset and 1 with early onset; One of the promoter common variants was also associated with age of onset, as well as the combined occurrence of two other promoter variants; Some of the identified SNPs could alter TTR splicing or disrupt/create transcription factor and miRNA binding sites	Several *TTR* non-coding variants were associated to age of onset and their putative effects could lead to alterations in gene expression that could contribute to the pathogenesis and clinical variability of the disease	[113]

Non-coding variants, such as SNPs and structural variants, influence gene regulation and expression and can alter transcription factor binding, splicing, and miRNA interactions. Studies have identified non-coding variants in the *TTR* gene linked to different phenotypes and disease onset, highlighting their impact on disease presentation. Further research is needed to fully understand their role in ATTRv amyloidosis.

## Fibril composition

As aforementioned, TTR proteolysis has been extensively studied as a mechanism that can drive amyloid formation [11]. This theory gained force due to the presence of different types of amyloid fibrils found in deposits. In fact, a study showed that proteolysis of TTR Ser52Pro, a variant that causes a severe form of amyloidosis, leads to the formation of a 49–127 fragment, which, when released in a physiological fluid agitation, quickly forms highly stable aggregates [114].

Although still under study, some authors have reported that amyloid fibril composition may have an influence on the disease phenotype.

There are two types of amyloid fibrils, type A and type B. Type A fibrils consist of a mixture of C-terminal fragments and full-length TTR, display weak affinity to congo red, and are weakly birefringent, whereas type B fibrils are solely composed of intact full-length TTR, have high affinity for congo red and are strongly birefringent [12]. In this study, Bergström’s team found both fibril types in Swedish ATTR Val30Met amyloidosis patients, yet type A fibrils were the only type found in individuals with ATTRwt amyloidosis, an amyloidosis that is frequently observed in older individuals and is mainly cardiac in nature [12].

Later, Ihse and peers analyzed fat tissue biopsies from 33 Swedish ATTR Val30Met amyloidosis patients and found that type A fibrils were associated with late disease onset and hypertrophied myocardium whereas type B fibrils were related to an earlier onset but no cardiac involvement [115].

Shortly after, a study by Koike and colleagues strengthened these findings by examining deposits from eight autopsied patients, all *TTR* Val30Met carriers, three early onset from endemic parts of Japan and five late onset from non-endemic regions of Japan [116]. They discovered that amyloid deposits in early-onset cases tended to be type B fibrils while those in late-onset cases were type A fibrils. Interestingly, an analysis of the cardiac deposits of the patients showed that in early-onset individuals the deposited TTR was mostly mutated, whereas in late-onset cases the deposits presented a significant amount of wild-type TTR. The authors hypothesized that these findings could support the idea that the mechanism behind amyloid deposition is similar to the one observed in ATTRwt amyloidosis.

In another study of Japanese ATTR Val30Met amyloidosis patients, an association with fibril type and age of onset was also found [117]. However, they did find a patient with type B fibrils and age of onset of 60 years of age. Cardiac assessment for type A fibril patients was only possible in one person; nonetheless, he did show a more severe cardiac involvement than type B patients, consistent with previous findings. Intriguingly, in the same study, the authors also assessed 63 non-Val30Met patients who all, except two, had type A fibrils and were unable to find a correlation between fibril type and phenotype. The authors ultimately postulate that fibrils with fragmented TTR might be the standard amyloid composition in ATTRv amyloidosis.

These studies uncover the amyloid fibril composition as a possible determinant in age of onset for the Val30Met variant.

It has also been found that, after liver transplantation, amyloids fibrils are mostly composed of wild-type TTR [118, 119] and one study has suggested that, not only is the heart more prone to incorporate wild-type TTR than other tissues, but type A fibrils incorporate higher amounts that type B fibrils [120]. This seems to corroborate previous findings, since late-onset patients typically have type A fibrils. In fact, Gustafsson and peers analyzed and confirmed this potential link as they discovered that patients with type A fibrils develop cardiomyopathy and heart failure (or their preexisting cardiomyopathy deteriorates) after liver transplantation, while the same is not seen in patients with type B fibrils, further suggesting that type A fibrils are more susceptible to continuous amyloid deposition from wild-type TTR [121].

It is thus fair to assume that TTR fibril composition does in fact play an important role, not only in phenotypic variation of the *TTR* Val30Met variant, but also in the determination of survival after liver transplantation.

Succinctly, amyloid fibril composition, classified as type A or type B, plays a significant role in the phenotype of ATTRv amyloidosis. Type A fibrils, containing C-terminal fragments and full-length TTR, are linked to a later-onset disease and more cardiac involvement, while type B fibrils, composed of intact TTR, are generally more associated with early-onset disease and less or no cardiac involvement. Studies suggest that fibril type influences disease progression and survival, but further research is needed to fully understand the prognostic and diagnostic implications of fibril composition in ATTRv amyloidosis.

## Epigenetic and environmental factors

Any process that alters gene expression and/or activity without modifying DNA sequences and can be inherited by daughter cells is considered an epigenetic modification. DNA methylation, histone modification, and miRNAs are three key epigenetic mechanisms [122].

Variability in phenotype and penetrance of ATTRv amyloidosis can conceivably be explained by epigenetic modifications especially when we consider monozygotic twins that carry the same variant yet display different clinical presentations [59–62]

In an epigenome-wide association study (EWAS) on 48 carriers (38 symptomatic and 10 asymptomatic) of *TTR* variants Val30Met, Phe64Leu, Ala120Ser, Ile68Leu, and Val122Ile and 32 controls, it was found that a CpG site in the Beta-secretase 2 (*BACE2*) gene was significantly hypomethylated in variant carriers [123]. BACE2 is known for cleaving APP in the brain, but is also present in peripheral tissues in inflammatory responses leading to the hypothesis that there might be a link between BACE2 and TTR-induced inflammation. They also found that the Val30Met variant disrupts a CpG site causing hypomethylation in carriers. This disrupted CpG site co-methylated with a CpG on the *B4GALT6* gene, a gene that showed significantly divergent methylation levels in symptomatic patients when compared to asymptomatic carriers and encodes an enzyme also related to inflammatory processes. They also report other CpG sites possibly related to disease symptoms (in genes *DSC2*, *DSG2* and *DSC3*) which the authors speculate might be involved in the formation of TTR deposits.

Posteriorly, a team performed an EWAS on *TTR* Val122Ile carriers of African descent to explore the possible association of methylation profile with heart disease and outpatient surgeries [124]. They found positive association between heart disease and methylation levels of genes *FAM129B*, *SKI*, *WDR27* and *GLS*; and between outpatient surgeries and methylation levels of *UBE2E3* and *SEC14LS* genes. Additionally, when analyzing differentially methylated functional modules, *ABCA1* was found to be significantly associated with heart disease. Importantly, the genes identified are involved in transport and clearance of amyloid deposits, cardiac fibrosis, and muscle tissue regulation, pathways that are important to the ATTRv amyloidosis pathogenesis.

As for miRNA, one study reported that Swedish, but not French or Japanese, carriers of the *TTR* Val30Met variant contained in their haplotype the T allele of the 3′UTR polymorphism rs62093482 [125]. The authors predicted that this allele could serve as a new miRNA binding site and hypothesized that it could lead to downregulation of the variant TTR synthesis which could explain Swedish low penetrance and late-onset disease. However, this theory was later refuted when a team found that the polymorphism had no effect on the regulation of variant *TTR* expression via miRNA [126].

Despite epigenetics being a promising field to help unravel the reasons behind clinical variability, further studies on this subject need to be carried out to further assess the role of epigenetic factors in ATTRv amyloidosis.

Regarding environmental factors, these are often referred to as a possible contributor to ATTRv amyloidosis variability, yet not many studies have focused on this.

One study reported an association between ATTRv amyloidosis and organic solvent exposure as well as with being a dressmaker, although these findings could be due to chance [127]. They also stated that history of prostatic hyperplasia, cholecystic disease, or appendectomy was a risk factor and hypothesized it could be due to anesthesia. It has also been suggested that gut microbiota could possibly play a role in amyloidogenesis [128]. Studies on mice carrying the *TTR* Val30Met variant further implied environmental factors in amyloidogenesis, as they reported that mice kept in specific pathogen-free conditions (i.e., minimum environmental interaction) exhibited substantially less amyloid deposition than mice maintained under conventional conditions [129, 130].

Epigenetic modifications, such as DNA methylation and miRNAs, can potentially impact the phenotype and penetrance of ATTRv amyloidosis. Studies have found differential methylation in genes associated with inflammation and amyloid deposition among variant carriers. Environmental factors, though less studied, may also contribute to disease variability and animal studies even suggest environmental conditions can affect amyloid deposition, indicating a complex interplay between genetics and environment in ATTRv amyloidosis.

## Other factors

Importantly, there are some factors that have been described in the literature that, despite being isolated reports, deserve to be mentioned.

The traditional model for gene expression in a diploid cell is by biallelic expression, where both alleles of a gene are expressed. On the other hand, monoallelic gene expression occurs when only one allele is actively transcribed while the other is silent [131]. Yordanova and colleagues analyzed the plasma and urine of 13 *TTR* Glu89Gln positive patients, with the aim to assess *TTR* transcription profile [132]. After analysis, the authors found a monoallelic expression signature in their cohort and proposed that this expression is age-related with wild-type TTR being expressed at a younger age subsequently shifting to variant TTR expression as the individual ages. This model could potentially explain why ATTRv amyloidosis is a disease of adult onset.

Somatic mosaicism, a phenomenon that has been implicated in disease states, is the presence of two genetically different populations of cells within a single individual as a result of a postzygotic variant [133]. In an interesting article, a large family with both healthy individuals and *TTR* Glu89Gln carriers was studied [134]. Analysis of peripheral blood, exfoliative buccal cells, and hair bulb cells from the *TTR* Glu89Gln carriers revealed some to have somatic mosaicism with reversion to normality in two cell types. Specifically, the *TTR* variant was found in the DNA of peripheral blood but not in that of buccal or hair bulb cells. This study raises the concern of testing for *TTR* variants in only one cell type since a false negative could easily be reported. It is unknown if this phenomenon has any impact in ATTRv amyloidosis severity or variability.

Monoallelic gene expression and somatic mosaicism are additional factors that may influence ATTRv amyloidosis. Monoallelic expression, where only one allele is transcribed, may shift from wild-type to variant *TTR* with age, potentially explaining adult-onset disease. Somatic mosaicism, the presence of different genetic populations within an individual, was observed in *TTR* Glu89Gln carriers, suggesting that variant testing in multiple cell types is necessary to avoid false negatives. The impact of these phenomena on disease severity and variability remains to be fully understood.

## Conclusions

Hereditary transthyretin-related amyloidosis (ATTRv amyloidosis) represents a complex and heterogeneous disease with significant phenotypic variability influenced by multiple factors. The genotype–phenotype correlation plays a crucial role in determining the clinical presentation and disease progression. Key variants like Val30Met, Val122Ile, and are associated with distinct phenotypes, affecting specific organs and systems. Age of onset further complicates the clinical landscape, with early- and late-onset presentations showing substantial differences in disease severity and affected organ systems.

Sex of the affected individual also impacts disease expression, with males often exhibiting earlier onset and more severe cardiomyopathy compared to females, potentially due to hormonal differences. The phenomenon of anticipation, particularly pronounced in maternal transmission of the variant, underscores the importance of parental inheritance patterns in disease progression.

Emerging evidence also suggests that non-coding variants, fibril composition, and epigenetic factors contribute to the phenotypic diversity observed in ATTRv amyloidosis. The distinction between type A and type B amyloid fibrils, and their respective impacts on disease manifestation and progression, highlights the importance of amyloid composition in understanding disease pathology. Epigenetic modifications, such as DNA methylation and miRNA interactions, and non-coding genetic variants can affect gene expression and/or regulation, potentially influencing the severity and presentation of the disease. These factors offer promising avenues for further research into the underlying mechanisms of this variability.

Environmental factors and isolated reports of phenomena like monoallelic expression and somatic mosaicism add additional layers of complexity to the disease.

In conclusion, all these findings underscore the necessity for comprehensive and multifaceted approaches in studying ATTRv amyloidosis. The intricate interplay between genetic, epigenetic, and environmental factors in ATTRv amyloidosis necessitates continued research to fully elucidate the mechanisms driving phenotypic variability. Such understanding is vital for improving diagnosis, genetic counseling, and the development of targeted therapeutic strategies, ultimately enhancing patient outcomes in this challenging disease.

## Future perspectives

The clinical variability of ATTRv amyloidosis presents a significant challenge to clinicians and researchers alike. Diagnosis, prognosis, and treatment of this disease, as well as its pathophysiology are complex. As such, ongoing research efforts are focused on improving our understanding of the underlying mechanisms that contribute to clinical variability in ATTRv amyloidosis, as well as developing new prognostic and therapeutic approaches. Hopefully, growing knowledge of the intricacies of ATTRv amyloidosis will lead to a better understanding of pathogenesis and disease course, as well as earlier and more accurate diagnosis and treatment.

## Data Availability

Not applicable.
